# On the Treatment of Deep Seated Dental Caries

**Published:** 1853-01

**Authors:** W. H. Dwinelle


AMERICAN JOURNAL

OF

DENTAL SCIENCE.

Vol. III. NEW SERIES—JANUARY, 1853. NO. 2.

### ARTICLE I.

*On the Treatment of Deep Seated Dental Caries.* By W. H. Dwinelle, M. D., D. D. S.

No. II.

In an article published in the April No. 1852, of the Journal, I took occasion to classify deep seated dental caries under three distinct heads.

Class I. Teeth, the nerves of which are only covered by softened bone, but which, otherwise, so far as can be ascertained, are healthy, or are capable of being restored to health.

Class II. Teeth, whose nerves are by accident, in excavating or otherwise, exposed—even lacerated, but which are healthy, or are capable of being restored to health.

Class III. Teeth, whose nerves are incapable of restoration, and whose destruction is inevitably required.

The article referred to, embraced all I intended to say in regard to class I. Class II and III, will form the subject of this article.

If the order of teeth referred to in my former article ranks first in importance, and if it is desirable to restore nature to the nearest approach to her original condition, teeth arranged under Class II, must rank next—indeed a tooth successfully treated of this class, is recognized by nature herself as being on an equality with any of its more fortunate neighbors, whether they rank under Class I, or have never been the subject of disease. She visits them with the same vitalizing energies, endows them with the same functions of production and of feeling, and links them to herself by the same delicate bond of union.

No fact is more clear, or more abundantly illustrated, especially in our own day, than, that nature, on the one hand, is endowed with the most wonderful recuperative energies; while on the other, she is capable of accommodating herself to the most extraordinary contingencies. Her principles and her actions are continually the subjects of new discovery.

A few years ago it was considered that the living brain could scarcely be touched without fatal consequences, but since large portions of it have been repeatedly taken away without material injury, and especially since the interesting experiments of a young man in Vermont, who by carelessness in blasting, suffered a crow-bar several feet long to be blown entirely through his head, carrying away large portions of his brain, and that too, without loss of consciousness, from the day of the explosion to this—since then, the old theory has exploded too!

A Lieut. Hamilton illustrates to us how a man can survive even after the spear of guerrilla has struck his breast, plunged through his lung and shown its bright blade at his back. Gen. Shields was a living witness that a man can sometimes be perforated in almost every vital part, and yet be stronger than death. With accupuncture needles, we pierce with impunity the blood vessels and nerves, the stomach and brain, and the French surgeons boast—even the heart! Had a man once asserted that a lost nose could be supplied from the forehead, a lip from the cheek, or that a deep, contracted cicatrix occupying one-fourth of the circuit of the neck, could be dissected out, and its place supplied with healthy living flesh from the arm or breast, such a man would have been "laughed to scorn;" and yet the rhinoplastic operation is almost daily performed. Even in our own day, to sever a tendon was to disorganize it and render it useless forever; now, many of the most important as well as frequent operations in surgery are founded upon the idea of severing the tendons in order that they may retract until the muscles have assumed a natural position. Nature, ever the willing handmaid of science, and never failing in her resources, pours into the void cartilaginous lymph, cementing the retracted ends together, and eventually consolidates it into a density and toughness equal to the strongest fibres of the system. Needles swallowed in infancy have traversed through the body, and in after years have come out at the top of the head or the extremity of the heel. Bones crushed to splinters, protrude through the flesh, they are forced back and replaced, osseous cement binds them together and the patient is restored. A bullet from some *careless* India-man's rifle lodges in an elephant's tusk instead of his heart, nature finding that she cannot dislodge the extraneous substance, goes to work to adapt and accommodate herself to the exigency. She first commences secreting ivory bone at the extreme surface of the tusk so as to enclose the cavity, and then gradually fills it up until the bullet is completely inlaid in its substance; so kindly does she conform herself to circumstances.

One of the angles of an incisor tooth is broken off by accident; immediately a corresponding angle in the pulp cavity is filled up by osseous deposit; so that while the nerve retracts so as to preserve an equidistant relation to the surface, it at the same time "*covers its retreat.*" The pulp of a tooth is accidentally laid bare in excavating a carious cavity, a concave cap of gold is nicely arched over it, and the tooth skillfully plugged. Months or years afterwards the inquisitive operator finding the tooth both living and healthy, is anxious to know in what manner nature has accommodated herself to the entertaining of a foreign substance in such close proximity to her most exalted and delicate tissues. He removes the stopping, the cap at first refuses to be displaced, a second effort separates it from the bottom of the cavity, when lo, he finds a white convex button of secondary dentine there, corresponding exactly to the inner surface of the cap, and even now, since the commencement of the writing of this series of articles, the new fact that a drill may perforate the living bone of the tooth and expose the nerve with impunity—even more, that the nerve may be actually severed, with the positive conviction that it will unite again, and all without hazarding the life of the tooth, gives us additional assurance that the field of physiological research is not yet fully explored, and that nature has still undiscovered facilities and powers, that shall yet dawn upon the path of science and investigation.

Perhaps I have dilated too largely upon this subject; my object in referring to these facts in this connection was, that we might gather encouragement from the continued march of the past, to press forward, regardless of the conservatism which is satisfied with the partial good we have attained, and impatient with progress for hoping for better things—to press forward, until nature shall yield to the persistence which demands the opening of the doors of her secret chambers, to the development of *all* of her resources.

Our profession it is true, is comparatively prosy and matter of fact, it is difficult to clothe it with beauty or attractiveness. And yet it has points of wonderful attraction, fields of surpassing beauty, which are continually opening to the light of science and patient inquiry. Let us then press forward to our sure reward.

In an article published in vol. 7, page 74, of the Journal, I expressed what was then my conviction that "to expose the nerve is always to subject ourselves to the necessity of removing it, * * * that it is ever unsafe to fill a tooth after the membrane of its nerve is broken," &c. &c. But my subsequent experience has created an entire revolution in my views upon the subject, as well as course of practice. I am now fully established in the belief that in a large majority of instances wherein the nerve has been exposed, even to bleeding, the tooth is capable of being restored to all its functional vitality, and to unimpaired and permanent usefulness. I know the profession is and has been largely, and I doubt not, honestly, divided on this subject. I am happy, however, for the sake of the profession, in believing that confidence is continually increasing in favor of the operation. Many who a few years ago were strong advocates for the removal of the nerve whenever or however exposed, are now enthusiastic and confident in aiding nature to recuperate herself.

Although my method of treating and filling over exposed nerves, may not be essentially different from that already familiar to the readers of the Journal, I trust I shall be excused for introducing it here, if for no other reason than to give consecutiveness and completeness to the treatment of the subject under consideration.

To do this more conveniently I shall subdivide this class of exposed nerves into subdivisions.

First, Healthy nerves which have been recently exposed in the removal of caries, or by other causes.

Second, Inflamed nerves, which are exposed.

The most careful operator, in excavating a carious tooth, is sometimes liable to expose the nervous pulp, the age of the patient, the character of the caries, and the situation of the tooth in the mouth, are all circumstances which sometimes combine to favor the accident.

In excavating a tooth unfavorably situated in the mouth of a young person, whose pulp cavity is comparatively near the surface, and the color of the carious portion corresponds to that of the healthy bone of the tooth, it does sometimes happen, without any preindication or forewarning, that suddenly, as if from some remote fibrous branch of the nerve, blood exudes into the cavity, when the laceration is more extensive the effusion of blood is more copious. At other times the nerve is exposed so as to be perceptible to the eye, or yielding to the touch of an instrument, and yet is not wounded so as to induce bleeding.

In treating a tooth whose nerve has been lacerated to bleeding, as above, it is my practice not to check its flow at once, but to let it continue until it has nearly exhausted itself, which will generally be within a few minutes from its commencement, then with a lock of cotton, saturated with concentrated spirits of camphor, combined with a small quantity of tannin, I wash out the cavity several times; this induces a retraction and collapse of the already exhausted blood vessels, while, in my opinion, it often, if not generally, stimulates the formative apparatus of the nerve to make immediate osseous deposit, to repair the violence which has been done. I have before spoken of the analogy between the teeth and that of other organs and parts of the system. In case of fractured bones, the system sometimes becomes indolent in its action, and refuses to secrete osseous deposit to cement the broken ends together, until the parts have been stimulated into life and action by external and internal treatment. Broken bones which have been separated for months, are sometimes readily united by laying them bare and scraping their extremities, when by irritation the sluggishness of the system is aroused to action, and an osseous floor supervenes. So with the teeth whose nerves are exposed; ossification may sometimes be induced by daily scraping lightly about the borders of the opening in the pulp cavity, protecting it in the interim with cotton, when it will soon be found that the exposed pulp is completely covered by secondary dentine.

After the bleeding of the nerve has once been arrested by the method prescribed, no fear need be entertained of its bleeding after the cavity is plugged. I have never known an instance to occur. The torn blood vessels seem to retract upon themselves, and heal at once.

Having fully prepared the cavity for the introduction of the gold, I now construct a concave cap of gold plate, and adapt it to its place at the bottom of the cavity; this is then dropped into a little dish of melted wax; on removing it, it will be found to be very thinly coated over with wax, so thin as scarcely to be perceptible. This gives it a non-conducting surface, while the wax about its edges, which come in contact with the bottom of the cavity, will enable you, with great certainty, to keep it in its place. The cavity being thoroughly dried, the cap is arched over the nerve, several rings of foil are first introduced and consolidated about the edges of the cap, when the operation is completed in the usual manner.

Teeth whose nerves are exposed, but do not bleed, are treated in precisely the same manner, with the difference, of course, that the operation is not retarded by bleeding. The camphorated spirits I always use in treating teeth of this character, it seems to combine the very desirable qualities of a sedative and a stimulant.

Dr. Harris, who has written extensively and clearly on this subject,* prefers arching the gold foil over the exposed nerves, thus constructing a cap at the time of the filling of the tooth. After properly preparing the nerve and cavity, he commences introducing the gold, "by placing the folds on one side of the cavity, and inserting fold after fold, without carrying those immediately over the exposed part of the pulp, to the bottom of the cavity, until every part, except a small space over the nerve, is thoroughly filled. The folds should be pressed so compactly one against the other, as to prevent their inner extremities from being forced against the exposed pulp in the consolidation of the protruding part of the filling. The gold should now be thoroughly condensed, and the surface finished in a suitable manner."

In many cases, this manner of capping the nerve is preferable to the other method, but the plate-cap is altogether preferable where it can be used.

It sometimes happens that teeth treated in the manner I have described, become highly inflamed and require treatment, but this is rarely the case, and whenever it does occur, they readily yield to the remedies I have prescribed in my former article on this subject.

I have placed this order of teeth first in my subdivision, because they are next in importance to those of Class I, the subject of my former article, and because success follows next in order, indeed it is rare that death of the pulp supervenes.

* Journal of Dental Science, Oct. 1851. Fourth edition of Principles and Practice of Dental Surgery, &c.

When it does not, the teeth thus treated give the same indications of vitality with those which have never been the subject of accident or disease. For a season they are often sensitive to warm and cold applications, but this soon wears away, except in some few instances, when months, or perhaps years afterwards, inflammation and suppuration supervene, in which case, the tooth may be retained by removing the nerve and filling the dental canal with gold.

In several cases, years afterwards, for the purpose of preparing for full sets of teeth on plate, I have removed teeth which I had successfully treated in the manner described, and have invariably found their nerves living and fully protected by a considerable deposit of secondary dentine immediately beneath the cap. I do not doubt but that all of the cases successfully treated, are alike favored in this respect.

From whatever cause this process of ossification is induced, it is desirable that it should be arrested when it has accomplished the end for which it was brought into action; this, however, is not always the case, we sometimes meet with cases wherein the nerve itself is partially ossified. While in London a few weeks ago, Mr. John Tomes, Surgeon Dentist to Middlesex Hospital, showed me among other curiosities in his extensive dental museum, the superior molar tooth of a middle aged person, whose tri-branched nerve was completely ossified, from the pulp cavity to its extremities. The tooth was not carious. It had been cracked open and the ossified pulp and nerve branches were exposed in relief, much resembling the living organ, with the difference, that it was semi-transparent and watery in appearance. The ossification, in this instance, had probably been induced by some violent concussion or jar upon the tooth, inducing a degree of inflammation of the nerve which did not spend itself until the entire organ was involved and destroyed.

We now come to the consideration of inflamed nerves which are exposed, but where inflammation has not so far proceeded as to produce disorganizing results; premising, also, that they have been recently exposed. If the inflamed nerve proves to be considerably congested, and is so situated that it can be distinctly seen, and readily approached with an instrument, guided by a careful hand, I have often found it advantageous, if it has not already been done, to carefully rupture its membrane, so as to induce bleeding. This cannot always be done, owing to its situation or the extreme sensitiveness of the patient, but when the engorged vessels are relieved by this depletion, I have found the nerve much more yielding to subsequent treatment. Tannin, spts. of camphor, and tannate of lead, for applications within the tooth, will be found of great value, while lancing about the neck of the tooth, together with the use of cathartics for the general system, will usually unite in soon reducing the inflamed organ to a condition of comparative health, when it may be capped and filled by the method already described.

In the event of these remedies being insufficient, the use of counter-irritants in the manner prescribed on page 435, vol. 2, of the Journal, (new series,) will generally produce the end desired. Should all efforts to subdue excessive inflammation fail, the only course left, is to remove the filling, destroy and extract the nerve and fill the canal to its extremity.

Teeth successfully treated as above, put on the same healthful appearance that distinguished them before they were diseased. Sometimes a relapse transpires, when the removal of the plug and nerve is indispensable to the retention of the tooth. I have occasionally removed some of these successfully treated cases, and have found the same deposit of new bone, as in the other cases cited. It is proper to add before dismissing this subject, that the teeth spoken of, under this last subdivision, more frequently fail under treatment than those under the first.

Since the discovery of "risodontrophy," I have performed that operation upon eleven teeth; two of them, upon the class of teeth last mentioned, and—upon this latter class—*after* I had filled them in the usual manner; and in all of the cases with entire success. The last two cases, were those wherein inflammation had supervened after they had been filled.

I have not had sufficient experience in the operation of "risodontrophy," to pass an unqualified verdict upon it; but I am inclined to think, that by its aid *all* exposed nerves, not already disorganized, may be restored to health, and the tooth to all of its vitality and usefulness. The new operation promises to be one of the most important discoveries ever made in operative dentistry. Should these expectations be realized, the discoverer or discoverers, will abundantly merit the unceasing gratitude of the profession, as well as all of those who are its subjects. It is another wonderful exemplification of the ability which nature possesses to adapt herself to great exigencies

Without presuming to put in *another claim* for priority of discovery, I trust I may be pardoned if I describe the manner in which I once accidentally, as well as successfully performed the operation which others have since developed and established.

About three years ago, Mr. C., a gentleman residing in this place, came to me to relieve him from excessive pain in his front teeth. On examination, I found the left superior frontal incisor, and also its neighbor, the lateral, the subjects of great inflammation. The teeth were loose, and the gums and membranes about them much swollen; both of them had been filled on their approximal surfaces. The nerve of the frontal had been dead for several years, and occasionally suppurated, formed an abscess at its extremity and discharged. It was my opinion at the time, that the nerve of the lateral incisor had recently been destroyed by the inflammation which then had the mastery over it, and that suppuration had already taken place.

Ever since I became acquainted with Dr. J. C. Flagg's operation of *tapping* teeth, whose nerves were dead, I had adopted it in treating teeth suffering from great inflammation of the periosteum from the death of the nerve, and threatened with abscess. I proceeded to treat these teeth in the same manner.

With a small drill I elevated the highest point of the festoon of the gum over the crown of the frontal tooth, and drilled into its nerve cavity; a flow of pus followed the instrument on its removal. I then proceeded to treat the lateral in the same manner; on reaching its nerve, the patient felt a sharp thrill of pain, and on withdrawing the instrument, several drops of red blood followed instead of pus. The operation upon each of the teeth afforded immediate relief from pain. Fearing the destruction of the nerve of the lateral would result as a consequence of its being punctured by the drill, I directed the patient to call again the next day, which he did, and to my great satisfaction, I found inflammatory symptoms had almost entirely subsided, and learned from my patient that he had experienced no return of pain. Several weeks after, Mr C. called at my office, and informed me that on that day, as a matter of curiosity, he had elevated the gum and introduced a bristle into the drill hole I had made, until it reached the nerve; that touching the nerve only gave him slight pain, and did not cause a flow of blood. I repeated the experiment with the same results. The tooth was entirely healthy and had not been painful since the first operation. About a year ago I again examined the lateral tooth, when I found the bottom of the drill hole entirely closed up; the tooth was still healthy. I never repeated the operation upon living teeth until recently, though I had often thought of reporting the above case as a novelty.

CLASS III will form the subject of an article in the next Journal.

(To be continued.)

